# Equity distribution of quality evaluation reports to doctors in health care organizations

**DOI:** 10.7717/peerj-cs.819

**Published:** 2022-01-21

**Authors:** Mahdi Jemmali, Loai Kayed B. Melhim, Abdullah Alourani, Md. Moddassir Alam

**Affiliations:** 1 Computer Science, Majmaah University, Zulfi, Riyadh, Saudi Arabia; 2 Computer Science, Mars Laboratory, Sousse, Tunisia; 3 Computer Science, Higher Institute of Computer Science and Mathematics, University of Monastir, Monastir, Monastir, Tunisia; 4 Computer Science, Hafr Batin University, Hafr Batin, Saudi Arabia

**Keywords:** Load balancing, Load work, Health care, Optimization

## Abstract

There are volumes of patient reports generated in any healthcare organization daily. The reports can be very lengthy or of few pages. Maintaining records of patients is essential for ensuring quality medical care. Doctors, apart from their routine activities, are also responsible to sort, examine and archive the generated reports. However, this process consumes doctors’ time, who are already hard-pressed for time. The objective of this study is to search for a method that can assign reports to doctors to ensure equitable and fair distribution of the overall workload. As a part of the solution, a mathematical model will be proposed to perform different developed heuristics. An experimental evaluation using different classes with a total of 2,450 different instances will be tested to measure the performance of the developed heuristics in terms of, elapsed time and gap value calculations. The clustering heuristics which is based on two groups is the best heuristic with 96.1% for the small instances and 98% for the big scale instances. The contribution of this work is based on employing dispatching rules with several variants; randomization approach, clustering methods; probabilistic method, and iterative methods approach to assign all given reports to doctors while ensuring the equitable distribution of the paper workload.

## Introduction

Doctors time in a healthcare organization is very precious. They spend time in face-to-face interaction with patients’ gathering information, doing administrative work such as paper tasks related to visits, quality, *etc*. Furthermore, a doctor spending more time with a patient is a requirement for value and efficiency in health care delivery. Assigning excess paperwork tasks has to be compensated with the allotted time for patient–doctor interaction. Further, it may affect their performance, elevate stress, lead to job dissatisfaction, may cause disruptive behavior and may also affect how doctors provide health care. This can also lead to medical errors, deficiencies in safety and low-quality health care services.

Thus managing the workload of doctors is very imperative. As doctors differ in the way they work based on their specializations, experience, job position and other functional or administrative considerations. Keeping this into consideration, fair distribution of doctor workload is very essential. Although studies have been conducted on optimization of doctor’s workload, to the best of the authors’ knowledge, none of the available studies has stressed the equity distribution of paper workload to doctors. Based on the above arguments, the present research aims at searching the problem of distributing paperwork load between doctors by developing a suitable algorithm to equitably distribute these paper works. The proposed study will have several positive implications such as suitable load distribution will ensure adequate time for patient–doctor interaction, helping the doctor to understand the patient disease state and prescribe better treatment and care. It will also allow doctors to spend their time updating their medical knowledge and devoting more time to clinical research.

Load balancing or equity distribution is defined as: the effective distribution of task loads among a group of workers, whether they are people, computers or applications (Jemmali & Alquhayz, 2020). Load balancing helps ensuring scalability and availability of services, which enable organization to provide more services with less efforts (Alharbi & Jemmali, 2020; Jemmali, 2019b; Alquhayz, Jemmali & Otoom, 2020). It is important to note that the term equitable distribution here does not mean equal distribution, but rather committing to a set of preferences during the distribution of tasks in a way that ensures fairness among the group of workers according to given preferences. The problem of maximization of the minimum aim for the load balancing on machines is treated in several research works (Haouari & Jemmali, 2008; Jemmali, Otoom & al Fayez, 2020).

The problem of load balancing was discussed by many researchers who aimed to present a suitable solution for this problem. For example, Jemmali (2019a) presented several approximate solutions for the problem of load balancing. While mathematical modeling of load balancing with a new proposed objective function that determines the indicator to select among the proposed heuristics was presented by Jemmali (2019b, 2021c). In the same context, the exact solution of the load balancing problem applied on the distribution of projects was presented in Alharbi & Jemmali (2020) and Jemmali (2021b). The machine-learning technique (decision tree) and the application of particle swarm optimization (PSO) presented by Bany Taha et al. (2020) can be utilized to extend some meta-heuristics for the load balancing problem. Another application of the load balancing approach is proposed in Jemmali (2021a) to develop new solutions for the smart parking problem.

The importance of load balancing emerged through the different areas that utilized this concept. Load balancing algorithms were presented for the aviation sector to be applied for gas turbines engine in Jemmali et al. (2019) and Jemmali, Melhim & Alharbi (2019). Recently, Alquhayz, Jemmali & Otoom (2020) utilized load balancing to present solutions for the dispatching files to different disc spaces. Moreover, equity distribution was implemented by Jemmali & Alquhayz (2020) where the authors proposed new network architecture by adding a new component called “scheduler”. This component is responsible to find the better schedule that ensures the best load balancing of packets to be forwarded to routers.

Also, Taha & Chowdhury (2020) proposed a new load balancing algorithm for E-Health system called (GALB) based on Genetic Algorithm (GA). To reduce latency and overhead while sending E-health encrypted data to the cloud, the tasks received by GALB algorithm will be distributed between main gateway and E-health nearby devices based on distance, task complexity and encryption policy length. In the area of health care, load balancing was employed by Friesen et al. (2011) to control the number of patients flow in six emergency departments within a mid-sized Canadian city. While Kolomvatsos, Panagidi & Hadjiefthymiades (2015) presented load balancing model that was oriented to distribute health care workers into a finite number of resources in a post-emergency scenario for both indoor or outdoor applications. Similarly, Stock et al. (2021) used load balancing to assist patients’ load across 12 hospitals.

Utilizing load balancing in healthcare organizations to derive equitable or fair distribution, was adopted by many researchers. For example, Schaus, Van Hentenryck & Régin (2009) presented constraint programming models to balance the workload of assigning daily tasks of newborn infant patients to nurses, while satisfying many other constraints. The presented approach produces an approximate solution with an accepted performance and it did not consider the different nurse qualifications that would affect the ability to perform the assigned tasks, which in turn can affect the performance of the whole system. The concept of load balancing in health organizations to balance the loads of health care assigned tasks and paperwork loads, was presented by Zhong et al. (2018) where the authors investigated the staffing proper ratio of doctors and support staff to provide the required quality of the health care services.

The impact of load balancing on doctors’ performance and on the quality of the delivered health care, was presented by many authors. For example, Christino et al. (2013) presented a discussion of an online survey, where they reported that paper workloads were recognized as potential barriers to doctors performance, provided services, health care quality, resident education and on the medical profession in general *i.e*. more workloads mean more interruptions and less productivity in health services. To reduce doctor’s unnecessary interruptions, Weigl et al. (2012) investigated the relation between workflow interruptions and doctors’ ability to manage their workload in a guarded and productive style. The objective of their study was to help doctors focus more on providing proper health care services by suggesting the smooth distribution of workloads within healthcare institutions. The smooth distribution may reduce unnecessary interruptions but it will not indicate clearly the amount of the workload assigned to each doctor. The answer to this was by attempted by Woolhandler & Himmelstein (2014), who proposed a method to quantify the number of administrative tasks and paper workload assigned to each doctor in the healthcare organizations. The researchers utilized an online-survey that was distributed to more than 4,720 doctors in the United States. The authors stated that doctors assigned administrative tasks are likely to have more paper workloads, which may decrease their career satisfaction and productivity. The reviewed researches showed that the most common complaints among doctors working on health organizations was the amount of paper workloads assigned to them. There are many proposed solutions for such problems, such as the equitable distribution of tasks especially the paper workloads or the load-balancing. Equitable distribution means the fair distribution or the load balancing of the assigned tasks among doctors in health organizations.

But no previous studies, till the writing of these lines utilize the load balancing techniques to equitably distribute paper workloads to achieve fair distribution of paper workloads among doctors in health organizations.

For the performance and safety of healthcare delivery, an equitable distribution of paper workload should be applied in health care organizations to give the doctors more space and more time, so that they have the ability to focus more on their clinical duties. The fair distribution of paperwork loads may be achieved by carefully distributing managerial workloads. In the proposed work, a set of main ideas will be presented, which are summarized as follows: The first idea, is to divide the doctors into groups based on the nature of the tasks assigned to them. The second idea, is the distribution of paperwork to the doctors in these groups, based on criteria that will be determined later. Finally, distribute the paperwork within the same group fairly among the group members so that the members of one group are equal in terms of the papers count for each of them. The nature of the paperwork assigned to each doctor must be taken into consideration in terms of specialization and in terms of the type of the required paperwork, as there is some paperwork that cannot be divided and requires completion by the same doctor who start processing it.

The rest of this paper will be organized as follows, “Methods” presents methods and the problem description, while the proposed heuristics will be presented in “Heuristics”. Discussion of the experimental results will be presented in “Experimental Results”. Finally, the conclusion will be presented in “Conclusion”.

## Methods

This section presents the main stages that are used to solve the problem of entity paper workload distribution, which will be discussed in details in the coming sections.

### Solution road map

A set of main ideas will be presented In this work, which can be summarized as follows: The first stage is to divide the doctors into groups based on the nature of the tasks assigned to them. The second stage is the distribution of paperwork to the doctors in these groups, based on criteria that will be determined later. Finally, distribute the paperwork within the same group fairly among the group members so that the members of one group are equal in terms of the number of papers required to be executed by each of them. The nature of the paperwork assigned to each doctor must be taken into consideration in terms of specialization and in terms of the type of the required report, as there is some paperwork that cannot be divided and requires completion by the same doctor who start processing it.

### Problem description

The problem of paper workload distribution is described as follows. The paper work load is in the form of reports, let *R* be the set of reports that will be assigned to different doctors. These reports will be processed by the assigned doctors then will be sent to the concerned committees for final confirmation. While, the number of independent reports is denoted by *n*_*r*_ and the number of doctors in the concerned health organization is denoted by *n*_*d*_. The set of doctors *D* is defined as follows $\{D_1,\cdots ,D_{n_d}\}$. The index of each doctor is specified by *i*. Each report is characterized by the estimated processing time. This time depends on the number of pages of the assigned report. In this research the report pages count will be used as an indicator for the time estimation calculations. Therefore, each report *j* is defined by its pages count and will be denoted by *np*_*j*_. All reports have pages counts that are defined in the set denoted by $\{np_1,\cdots ,np_{n_r}\}$. The total number of pages assigned to each doctor *i* is denoted by $N_{D_i}$, while the number of pages assigned to doctor *i* when report *j* is assigned is defined as *Cn*_*j*_. The objective of this research is to ensure the equity distribution of reports pages between doctors. To achieve this goal, this work should present an indicator that can calculate the efficiency of the distribution process. This indicator will be known as *T* and will be expressed in Eq. (1). *T* is the gap value between each total number of pages assigned to every doctor *i* and the minimum number of the assigned pages. This gap is the measure of the equity distribution process. A large gap value indicates that the equity distribution is not reached, while a small gap values indicates that a fair distribution is reached. Thus, the objective of this work, is to find a schedule that minimizes the gap values *i.e*. The goal is to minimize *T*.

(1) $$T = \sum\limits_{i = 1}^{{n_d}} ({N_{{D_i}}} - {N_{min}}).$$

where *N*_*min*_ is the minimum number of assigned pages. $N_{} = min_{i = \{1,\ldots ,n_d}\}N_{D_i}$. Table 1 represents all notations used in this paper.

**Table 1:** Notations used in this paper with their definitions.

Symbols	Explanation
*R*	set of reports that will 104 assigned to different doctors
*n*_*r*_	number of independent reports
*n*_*d*_	number of doctors in the concerned health organization
$D = \{D1,\cdots,D_{n_d}\}$	set of doctors
*i*	index of each doctor
*j*	index of each report
*np*_*j*_	number of pages
*N_D_*_*i*_	total number of pages assigned to each doctor *i*
*Cn*_*j*_	number of pages assigned to doctor *i* when report *j* is assigned
*N*_*min*_	minimum number of assigned pages
*limit*	number of iteration
H*	the minimum *T* values returned after the execution of all heuristics *H*_1_ to *H*_8_.
*H*	represents the *T* values returned by the heuristics *H*_1_ to *H*_8_.
*Perc*	is the percentage for each heuristic to reach H*.
$G = \displaystyle{{H - {H^*}} \over H}$	if *H* = 0 then *G* = 0. gap value between H* and *H*
*Ag*	average of *G* for a fixed number of instances.
*Time*	running time in seconds, or the result of “-” if the time is less than 0.001 s.

**Example 1** *Let n*_*r*_ *=* 7 and *n*_*d*_ = 2. *The number of pages for each report is displayed in Table 2*.

**Table 2:** 7-2 instance of pages’ number reports.

*j*	1	2	3	4	5	6	7
*np*_*j*_	20	25	14	12	30	15	10

**Figure 1:**
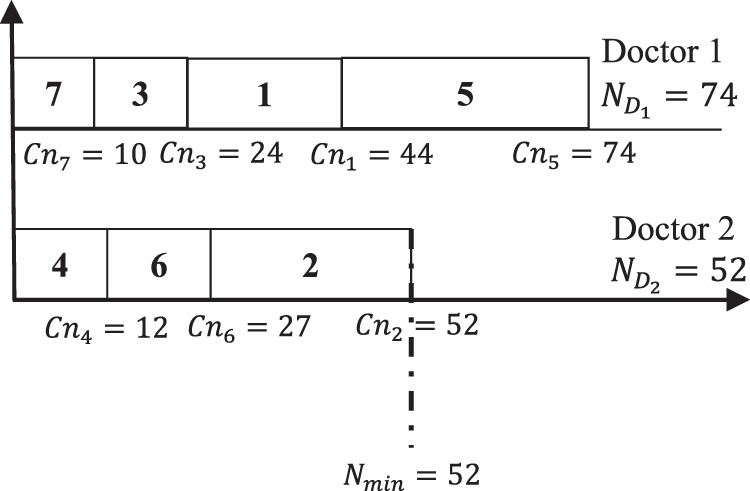
7-2 reports-doctors finishing time distribution.

Figure 1 presents a schedule to show the reports assignment to each doctor.

**Figure 2:**
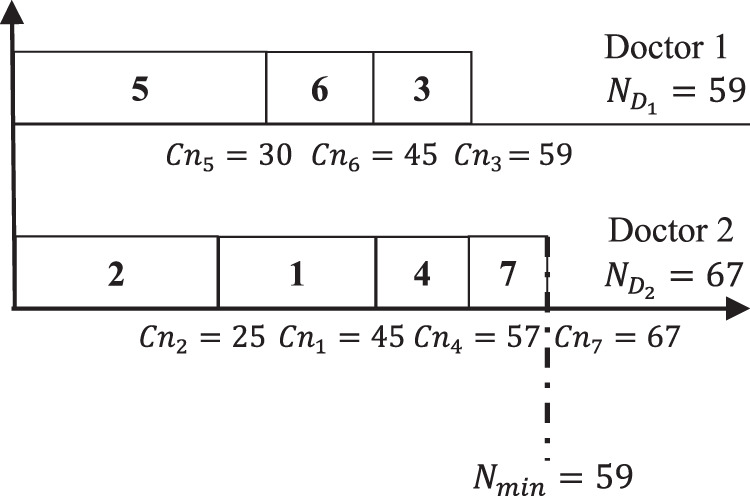
Max–min ameliorated schedule.

Figure 1 shows that the minimum total number of pages $(N_{D_2} = 52)$ is assigned to Doctor 2. However, the maximum total number of pages $(N_{D_1} = 74)$ is assigned to Doctor 1. The gap of total number of pages *T* between doctors, is calculated by applying Eq. (1) as $T = \sum\nolimits_{i = 1}^{{n_d}} ({N_{{D_i}}} - {N_{min}}) = (74 - 52) + (52 - 52) = 22$. The main objective is to develop another schedules that should ameliorate the gap between the doctors paperwork. For the same example if another schedule is applied then a different scenario will be obtained as shown in Fig. 2.

Figure 2 shows that the minimum total pages $(N_{D_1} = 59)$ is assigned to Doctor 1, while the maximum total number of pages $(N_{D_2} = 67)$ is assigned to Doctor 2. Thus, the new calculated gap between doctors after applying Eq. (1) is (67 − 59) + (59 − 59) = 8. Comparing with scheduling result shown in Fig. 1, it is clear that the schedule in Fig. 2 is better that schedule presented in Fig. 1. The gained gap between the two schedules is 22 − 8 = 14.

Therefore, improving the calculated gap will enhance the fair distribution of reports to different doctors, which will gain more free time to doctors, more resting time and more time to deliver improved health care services to patients. Considering the number of processed reports throughout the doctor’s service period and the number of doctors involved in this process, will demonstrate the amount of time that can be saved and the benefits that can be achieved for doctors, patients and for the health care organizations, if proper scheduling is been adopted. This goal motivates us to present this research and to develop the required heuristics to achieve this goal.

## Heuristics

In order to solve the proposed problem several heuristics is developed and will be presented in this section. The first two heuristics *H*_1_ and *H*_2_ are based on the dispatching rules. The heuristic *H*_3_ is based on the randomization of the doctor choice, where the choice of the doctor will be performed randomly. Heuristic *H*_4_ will apply the same steps of *H*_3_, except that it will exclude the doctor whose report *j* − 1 has been assigned. While *H*_5_ is obtained after excluding the doctor who has the most workload of reports. For heuristic *H*_6_, it will be responsible for calling *H*_3_, *H*_4_ and *H*_5_ heuristics with iteration and then selecting the best of the returned results. The iteration process will be performed separately for the invoked heuristics *H*_3_, *H*_4_ and *H*_5_. Finally, heuristic *H*_8_ is constructed based on the clustering method. Which means that the classification of reports into groups give us the possibility to find a good solution.

For iteration based heuristics, the choice of 2,000 times was selected based on an experimental test. Indeed, we tested the heuristics for several iterations number. When the iteration number is larger than 2,000, it consumes more time without any significant effect on the results. However, if the iteration number is less than 2,000, for example (50–1,000), this will consume less time, but with a remarkable difference in the results when compared with the 2,000 iterations.

### Non-increasing order heuristic (*H*_1_)

This heuristic is based on the arranging of all given reports in a non-increasing order, according to their number of pages. After that, assign the report that has the biggest number of pages count to the doctor who has the minimum number of total pages’ count. The complexity of *H*_1_ heuristic is *O*(*nlogn*).

### Non-decreasing order heuristic (*H*_2_)

This heuristic is based on arranging all the given reports in a non-decreasing order according to their number of pages. After that, assign the report that has the lowest number of pages count to the doctor who has the minimum number of total pages’ count. The complexity of *H*_2_ heuristic is *O*(*nlogn*).

### Iterative random doctor choice without excluding heuristic (*H*_3_)

This heuristic adopts the randomization method. In this heuristic, when there is a need to assign a report, the required doctor to process that report will be chosen randomly. After finishing the schedule of all reports, an iteration loop will be executed to repeat the choice of doctors, this will generate a different schedule for each iteration. For this type of heuristics, three variants are considered. The first variant is the choice of the report to be scheduled according to report index. The second variant is the choice of the report to be scheduled according to the increasing order of the report’s pages count. The third variant is the choice of the report to be scheduled according to the decreasing order of the report’s pages count. After the execution of these variants the best solution is selected. In practice the iteration loop is executed 2,000 times. The complexity of *H*_3_ heuristic is *O*(*n*^2^).

The function random(*x*,*y*) returns a random integer between *x* and *y*. Schedule(*r*,*j*) is the function that assigns the report *j* to the doctor *r*. While, Incs() is the function that sorts the given reports in an increasing order based on their number of pages. Moreover, Decs() is the function that sorts the given reports in a decreasing order based on their number of pages. In practice the number of iterations is fixed with a value of *limit* = 2,000.

This heuristic is denoted by *H*_3_ and the related heuristic is described in Algorithm 1.

**Algorithm 1:** Iterative random doctor choice without excluding heuristic (*H*_3_).

1: Initialize *k* = 1 and *it* = 1
2: **for** (*k* = 1 to 3) **do**
3: **if** (*k* = 2) **then**
4: Incs(*R*)
5: **else if** (*k* = 3) **then**
6: Decs(*R*)
7: **end if**
8: **for** (*it* = 1 to *limit*) **do**
9: **for **(*j* = 1 to *n*_*r*_) **do**
10: Set *r* = random(1, *n*_*d*_)
11: Call schedule(*r*, *j*)
12: **end for**
13: Calculate $T_k^{it}$
14: **end for**
15: Calculate ${T_k} = \mathop {\min }\limits_{1 \le it \le limit} T_k^{it}$
16: **end for**
17: Calculate $T = \mathop {\min }\limits_{1 \le k \le 3} {T_k}$
18: Return *T*

### Iterative random doctor choice excluding the last doctor heuristic (*H*_4_)

This heuristic applies the same ideas described in *H*_3_. However, there is a difference in the choice of the assigned doctor. At a given time *t* when there is a need to assign the report *j*, select randomly any doctor from the list of the available doctors excluding the doctor whose report *j* − 1 has been assigned. The three variants described in *H*_3_ are also applied in this heuristic. After execution of these variants the best solution is selected. In practice the iteration loop is done 2,000 times. The complexity of *H*_4_ heuristic is *O*(*n*^2^).

### Iterative random doctor choice excluding the most loaded doctor heuristic (*H*_5_)

This heuristic applies the same ideas described in *H*_3_ with a difference the choice of the assigned doctor. At a given time *t* when there is a need to assign the report *j*, select randomly any doctor from the list of available doctors excluding the doctor who has the highest load. The three variants described in *H*_3_ are also applied in this heuristic. After execution of these variants the best solution is selected. In practice the iteration loop is done 2,000 times. The complexity of *H*_5_ heuristic is *O*(*n*^2^).

### Iterative random doctor choice heuristic (*H*_6_)

This heuristic is based on the iterative method. For each iteration and for each type of report pages sorting, apply the heuristic *H*_3_, *H*_4_, and *H*_5_ with some modifications. These modifications are based on the functions of these heuristics as described below. The best obtained value is returned. The complexity of *H*_6_ heuristic is *O*(*n*^2^).

*H*_3_() is the function of the heuristic *H*_3_ without the 2,000 iterations loop. *H*_4_() is the function of the heuristic *H*_4_ without the 2,000 iterations loop. *H*_5_() is the function of the heuristic *H*_5_ without the 2,000 iterations loop. *V*() is the value returned by the applied heuristic. Indeed, $V_k^{it}$ represents the value returned by *H*_*k*_() for the iteration *it*. In practice the number of iterations is fixed to *limit* = 2,000. The instructions of the heuristic *H*_6_ are described in Algorithm 2.

**Algorithm 2:** Iterative random doctor choice algorithm (*H*_6_).

1: **for** (*k* = 1 to 3) **do**
2: **if** (*k* = 2) **then**
3: Incs(*R*)
4: **else if** (*k* = 3) **then**
5: Decs(*R*)
6: **end if**
7: **for** (*it* = 1 to *limit*) **do**
8: Call *H*_3_() and calculate $V_3^{it}$
9: Call *H*_4_() and calculate $V_4^{it}$
10: Call *H*_5_() and calculate $V_5^{it}$
11: **end for**
12: Calculate ${T_k} = \mathop {\min }\limits_{3 \le i \le 5} (\mathop {\min }\limits_{1 \le it \le limit} V_i^{it})$
13: **end for**
14: Calculate $T = \mathop {\min }\limits_{1 \le k \le 3} {T_k}$
15: Return *T*

### Randomly repeating iteratively doctor choice heuristic (*H*_7_)

This heuristic is based on the iterative method. For each iteration and for each type of report pages sorting, apply the heuristics *H*_3_, *H*_4_, and *H*_5_ with the modifications that are based on the functions of these heuristics as described below. Compared to *H*_6_ the difference will be in the nature of performing the loop iterations. Indeed, for *H*_6_, a loop of *limit* iteration is performed on all heuristics *H*_3_(), *H*_4_() and *H*_5_() for 1 time. However, for *H*_7_, the iteration will be performed *limit* iterations for each heuristic separately. The best value is returned. The complexity of *H*_7_ heuristic is *O*(*n*^2^).

$H_3^*()$ is the function of the heuristic *H*_3_ with only one sorting choice. $H_4^*()$ is the function of the heuristic *H*_4_ with only one sorting choice. $H_5^*()$ is the function of the heuristic *H*_5_ with only one sorting choice. *W*() is the value returned by the applied heuristic. Indeed, $W_k^{it}$ represents the value returned by *H*_*k*_() for the iteration *it*. In practice the number of iterations is fixed by *limit* = 2,000. The instructions of heuristic *H*_7_ are described in Algorithm 3.

**Algorithm 3:** Randomly repeating iteratively doctor choice heuristic (*H*_7_).

1: **for** (*k* to 3) **do**
2: **if** (*k* = 2) **then**
3: Incs(*R*)
4: **else if** (*k* = 3) **then**
5: Decs(*R*)
6: **end if**
7: **for** (*it* = 1 to *limit*) **do**
8: Call $H_3^*()$ and calculate $W_3^{it}$
9: **end for**
10: **for** (*it* = 1 to *limit*) **do**
11: Call $H_4^*()$ and calculate $W_4^{it}$
12: **end for**
13: **for** (*it* = 1 to *limit*) **do**
14: Call $H_5^*()$ and calculate $W_5^{it}$
15: **end for**
16: Calculate ${T_k} = \mathop {\min }\limits_{3 \le i \le 5} (\mathop {\min }\limits_{1 \le it \le limit} W_i^{it})$
17: **end for**
18: Calculate $T = \mathop {\min }\limits_{1 \le k \le 3} {T_k}$
19: Return *T*

### Clustering-based heuristic (*H*_8_)

This heuristic is based essentially on the classification method. This research identifies two groups of reports. These groups are denoted by *G*_1_ and *G*_2_. The first phase of this heuristic is to choose the reports sorting method. The sorting methods will be in three variants. The first variant will consider to assign a report directly after it is initiated. The second variant will consider to sort the reports ascending based on their number of pages. The third variant will consider to sort the reports descending based on their number of pages. The second phase of this heuristic is the construction of *G*_1_ and *G*_2_ groups. Initially *G*_1_ and *G*_2_ are empty.

The third phase is to initiate *G*_1_ and *G*_2_ groups by distributing the set of reports *R*. The first step is to choose the first report and to distribute it to either *G*_1_ or *G*_2_, then the second report will be sent to the most available group between *G*_1_ and *G*_2_. The most available group will be the group that has the minimum pages count. After that assign the next report to the most available group and so on until all reports in *R* are distributed.

The fourth phase is the scheduling of the reports to the available doctors. This phase is based on the randomly selection between *G*_1_ and *G*_2_, by generating a probability *α* to select a report from the two groups. The selected report will be assigned to the doctor who has the minimum total number of pages. This procedure will be repeated many times. In practice the number of iterations is fixed to *limit* = 2,000.

Finally, the heuristic is repeated again with another sorting method variant and iterated for *limit* = 2,000 times, after all sorting variants are chosen the best final results are considered. The complexity of *H*_8_ heuristic is *O*(*n*^2^).

**Algorithm 4:** Randomly repeating iteratively doctor choice heuristic (*H*_7_).

1: **for** (*k* = 1 to 3) **do**
2: **if** (*k* = 2) **then**
3: Incs(*R*)
4: **else if** (*k* = 3) **then**
5: Decs(*R*)
6: **end if**
7: Determine *G*_1_ and *G*_2_
8: **for** (*it* = 1 to *limit*) **do**
9: **for** (*j* = 1 to *n*_*r*_) **do**
10: *r* =random(1,2)
11: **if** (*r* = 1) **then**
12: schedule the first report in *G*_1_
13: **else**
14: schedule the first report in *G*_2_
15: **end if**
16: **end for**
17: Calculate $T_k^{it}$
18: **end for**
19: Calculate ${T_k} = \mathop {\min }\limits_{1 \le it \le limit} T_k^{it}$
20: **end for**
21: Calculate $T = \mathop {\min }\limits_{1 \le k \le 3} {T_k}$
22: Return *T*

## Experimental results

This section describes the performance of the developed heuristics. All heuristics proposed in this paper were implemented in Microsoft Visual C++ and executed on an Intel(R) Core (TM) i5-3337U CPU @ 1.8 GHz and 8 GB RAM. Several classes of instances were being tested in this paper to measure the performance of the developed heuristics. These classes were generated by methods derived from Jemmali (2019b). The number of pages *np*_*j*_ for each report are generated by using two different distributions. The first distribution is the uniform distribution which is denoted by *U*() and the normal distribution that is denoted by *N*().

The generated classes are as follows:

- Class A: *np*_*j*_ ∈ *U*(10, 20).
- Class B: *np*_*j*_ ∈ *U*(20, 30).
- Class C: *np*_*j*_ ∈ *U*(10, 30).
- Class D: *np*_*j*_ ∈ *N*(20, 5).
- Class E: *np*_*j*_ ∈ *N*(20, 10).

Two types of instances are adopted for the experimental part. The small instances type and the big scale type. Both types were used to generate the *np*_*j*_ for all the classes above. For the small type instances, several values for the pair (*n*_*r*_, *n*_*d*_) were selected as Table 3 illustrates.

**Table 3:** Generation of (*n_r_*, *n_d_*).

*n*_*r*_	*n*_*d*_
5	2, 3, 4
7, 10	2, 3, 4, 5, 6
15, 20, 25, 30, 35	3, 5, 7, 10

For each tuple (*n*_*r*_, *n*_*d*_, *class*), this work will generate 10 different instances of the number of pages. Therefore, the total number of the generated instances will be (3 + 2 × 5 + 5 × 4) × 10 × 5 = 1,650. For the big scale instances, generate 800 instances as detailed in the end of this section. So, in total 2,450 instances were tested. Now, we start the analyses of results related to the 1,650 small instances.

To assess the performance of the proposed heuristics, the metrics *Perc*, *Ag* and *Time* are defined in Table 1. Hereafter, the analyses of the experimental results are concerning the small instances type.

The overall performance of the developed heuristics is shown in Table 4. As it can be noticed from the table, the best performance was obtained by the heuristic *H*_8_. The obtained percentage for this heuristic was 96.1% which is the highest among all heuristics. The gap calculations for *H*_8_ gained zero results for most of the instances except for 64 instances out of 1,650 leading to an average gap of 0.02, while the elapsed time was 0.006 s. For heuristic *H*_7_ the percentage was 59.1% with an average gap of 0.29 and an average time of 0.021s, which makes it the second best heuristic. However, the heuristic that has the worst performance measurements was *H*_2_, with the highest average gap of 0.74 and the lowest percentage of 0.8%.

**Table 4:** Overall performance of all heuristics.

	*H*_1_	*H*_2_	*H*_3_	*H*_4_	*H*_5_	*H*_6_	*H*_7_	*H*_8_
*Perc*	45.2%	0.8%	48.1%	37.5%	50.7%	57.6%	59.1%	96.1%
*Ag*	0.39	0.74	0.40	0.45	0.34	0.30	0.29	0.02
*Time*	–	–	0.003	0.005	0.014	0.022	0.021	0.006

Table 5 represents the variation of *Ag* and *Time* according to *n*_*r*_ for all developed heuristics. This table shows that the highest gap was obtained for heuristic *H*_2_ when *n*_*r*_ = 35, while the lowest gab of 0 was obtained by *H*_8_, *H*_7_, *H*_6_ and *H*_3_ for *n*_*r*_ = 5 and *n*_*r*_ = 7. As it can be noticed from Table 5, most of the heuristics perform well when *n*_*r*_ = 5, *n*_*r*_ = 7 and *n*_*r*_ = 10 and the performance values was close to each other, but for *n*_*r*_ = 35 the developed heuristics showed a different performance. So, based on gap calculations heuristic *H*_2_ showed the worst performance while heuristic *H*_8_ showed the best performance even for high *n*_*r*_ values such as 30 and 35. Based on time calculations heuristic *H*_6_ showed the worst performance at *n*_*r*_ = 35 then heuristic *H*_7_ also at *n*_*r*_ = 35. For heuristics *H*_3_ to *H*_7_ time increases as *n*_*r*_ is increases. The remarkable performance was shown by heuristic *H*_8_ as it can be noticed by the results shown in Table 5 the gap values = 0 and the running time was 0.001 for all *n*_*r*_ values.

**Table 5:** *Time* and *Ag* variations according based on *n_r_* for all heuristics.

		*n*_*r*_							
		5	7	10	15	20	25	30	35
*H*_1_	*Ag*	0.14	0.21	0.34	0.27	0.49	0.64	0.42	0.57
	*Time*	–	–	–	–	–	–	–	–
*H*_2_	*Ag*	0.46	0.51	0.73	0.73	0.84	0.86	0.89	0.90
	*Time*	–	–	–	–	–	–	–	–
*H*_3_	*Ag*	0.00	0.00	0.08	0.42	0.61	0.67	0.73	0.74
	*Time*	0.001	0.002	0.002	0.003	0.004	0.004	0.005	0.006
*H*_4_	*Ag*	0.24	0.21	0.29	0.37	0.57	0.62	0.67	0.69
	*Time*	0.002	0.003	0.003	0.004	0.005	0.006	0.007	0.009
*H*_5_	*Ag*	0.10	0.13	0.10	0.31	0.46	0.53	0.58	0.59
	*Time*	0.003	0.005	0.006	0.012	0.015	0.019	0.023	0.027
*H*_6_	*Ag*	0.00	0.00	0.01	0.29	0.46	0.49	0.60	0.60
	*Time*	0.006	0.008	0.011	0.019	0.025	0.030	0.036	0.041
*H*_7_	*Ag*	0.00	0.00	0.00	0.28	0.43	0.49	0.59	0.60
	*Time*	0.007	0.008	0.010	0.018	0.024	0.029	0.035	0.040
*H*_8_	*Ag*	0.00	0.00	0.00	0.00	0.00	0.00	0.00	0.00
	*Time*	0.001	0.001	0.001	0.001	0.001	0.001	0.001	0.001

Table 6 represents the variation of *Ag* and *Time* according to *n*_*d*_ for all developed heuristics. The 0 gap values were obtained by many heuristics at different *n*_*d*_ values, even heuristic *H*_1_ had a zero gap value at *n*_*d*_ = 6. But in general as the value of *n*_*d*_ increases, the gap value and the elapsed time increases for all heuristics except for heuristic *H*_8_, where it again shows a better performance values than the rest of the heuristics even with large *n*_*d*_ values. So, based on gap values heuristic *H*_2_ shows the worst performance and heuristic *H*_8_ showed the best performance. Heuristics *H*_1_ and *H*_2_ have the minimum average time, while heuristic *H*_8_ showed an average time of 0.001 for all *n*_*d*_ values.

**Table 6:** *Time* and *Ag* variations according based on *n_d_* for all heuristics.

		*n*_*d*_						
		2	3	4	5	6	7	10
*H*_1_	*Ag*	0.52	0.54	0.18	0.33	0.00	0.51	0.28
	*Time*	–	–	–	–	–	–	–
*H*_2_	*Ag*	0.80	0.83	0.51	0.80	0.36	0.81	0.70
	*Time*	–	–	–	–	–	–	–
*H*_3_	*Ag*	0.00	0.19	0.05	0.62	0.01	0.72	0.69
	*Time*	0.002	0.003	0.002	0.004	0.002	0.005	0.005
*H*_4_	*Ag*	0.73	0.18	0.11	0.58	0.04	0.68	0.68
	*Time*	0.003	0.006	0.002	0.005	0.002	0.006	0.006
*H*_5_	*Ag*	0.44	0.03	0.00	0.49	0.01	0.62	0.65
	*Time*	0.004	0.008	0.005	0.012	0.006	0.020	0.031
*H*_6_	*Ag*	0.00	0.02	0.00	0.48	0.00	0.61	0.63
	*Time*	0.009	0.017	0.008	0.021	0.011	0.031	0.043
*H*_7_	*Ag*	0.00	0.01	0.00	0.46	0.00	0.61	0.63
	*Time*	0.008	0.017	0.007	0.020	0.010	0.029	0.041
*H*_8_	*Ag*	0.00	0.00	0.00	0.00	0.00	0.00	0.00
	*Time*	0.001	0.001	0.001	0.001	0.001	0.001	0.001

Table 7 illustrates the variation of *Ag* and *Time* according to *Class* for all developed heuristics. As was noticed in the results shown in Tables 6 and 5, the performance measurement of heuristic *H*_2_ showed the worst performance compared to the rest of the heuristics, heuristic *H*_2_ returned the highest average gap when class = 5 with a value equal to 0.83. The rest of the heuristics showed comparable performance for all used classes except for heuristic *H*_8_. The average gap values ranged from 0.23 to 0.52, the highest gap values were obtained for classes 3 and 5. But for heuristic *H*_8_, again it shows the best performance measures with average gap value = 0.01 with an elapsed time of 0.001 s.

**Table 7:** *Time* and *Ag* variations according based on *Class* for all heuristics.

		*Class*				
		1	2	3	4	5
*H*_1_	*Ag*	0.34	0.24	0.43	0.47	0.45
	*Time*	0.000	0.000	0.000	0.000	0.000
*H*_2_	*Ag*	0.72	0.61	0.79	0.76	0.83
	*Time*	0.000	0.000	0.000	0.000	0.000
*H*_3_	*Ag*	0.39	0.35	0.40	0.40	0.44
	*Time*	0.003	0.003	0.003	0.003	0.003
*H*_4_	*Ag*	0.43	0.39	0.48	0.45	0.52
	*Time*	0.005	0.005	0.005	0.005	0.005
*H*_5_	*Ag*	0.32	0.26	0.38	0.36	0.40
	*Time*	0.014	0.013	0.014	0.014	0.013
*H*_6_	*Ag*	0.27	0.25	0.32	0.30	0.35
	*Time*	0.022	0.022	0.022	0.022	0.022
*H*_7_	*Ag*	0.26	0.23	0.31	0.30	0.34
	*Time*	0.021	0.021	0.021	0.021	0.021
*H*_8_	*Ag*	0.01	0.01	0.01	0.01	0.01
	*Time*	0.001	0.001	0.001	0.001	0.001

The experimental results show that the maximum average gap reaches 0.97 for *H*_2_ when (*n*_*r*_ = 20, *n*_*d*_ = 3), (*n*_*r*_ = 25, *n*_*d*_ = 3), and (*n*_*r*_ = 35, *n*_*d*_ = 3). The 0.00 average gap was obtained by *H*_3_, *H*_6_, *H*_7_ and *H*_8_ for *n*_*r*_ = 5 and *n*_*r*_ = 7 for all *n*_*d*_ values, but when *n*_*r*_ values begin to be larger than 10 the average gap results, start to increase for most of the heuristics except for *H*_8_ where the heuristic shows a 0.00 average gap results for all *n*_*r*_ and *n*_*d*_ values.

Hereafter, *Ind* is the index of each pair (*n*_*r*_, *n*_*d*_). In total we have 33 different pairs.

**Figure 3:**
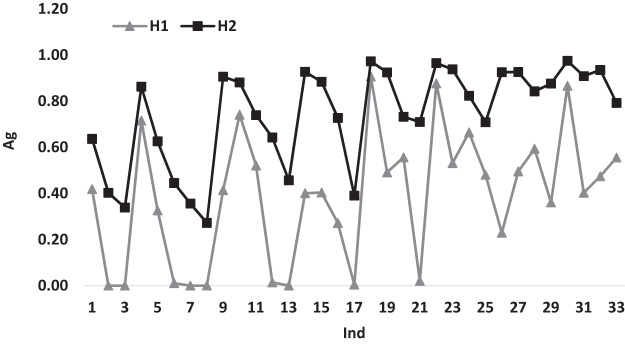
The average gap for *H*_1_ and *H*_2_ according to *Ind*.

Figure 3 illustrates the variation of the average gap for heuristics *H*_1_ and *H*_2_ according to *Ind*. The behavior of the gap variations for both heuristics shows a similar behavior for most of *Ind*. This behavior is expected, because of the similarity in the way the two heuristics work. Despite the similar behavior, *H*_1_ performs better than the *H*_2_, there are some points where *H*_1_ gap values reaches 0.00 while *H*_2_ gap values never reaches 0.00 for all *Ind*.

**Figure 4:**
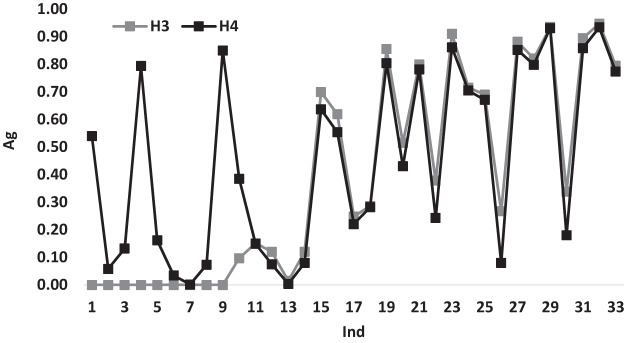
The average gap for *H*_3_ and *H*_4_ according to *Ind*.

Figure 4 illustrates the variations of the average gap for heuristics *H*_3_ and *H*_4_ according to *Ind*. From the figure it can be noticed that *H*_3_ and *H*_4_ shows similar behavior variations starting from *Ind* = 9, while before that point *H*_4_ has 0.00 gap values. The similar behavior is expected as both heuristics, mostly use the same technique most of the time with a difference in the choice of the assigned doctor.

**Figure 5:**
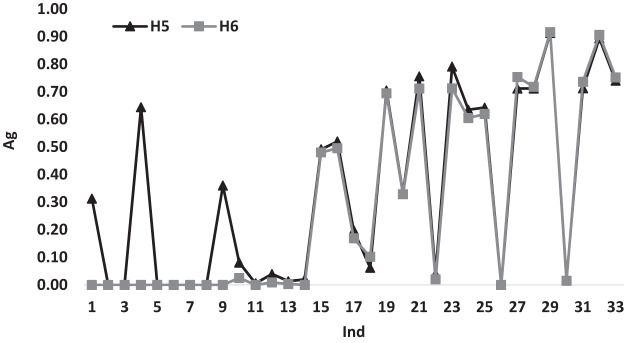
The average gap for *H*_5_ and *H*_6_ according to *Ind*.

Figure 5 illustrates the variations of the average gap for heuristics *H*_5_ and *H*_6_ according to *Ind*. It can be noticed that *H*_6_ has a better performance measures with a gap value of 0.00 till *Ind* = 14 and beyond this point, both heuristics have similar behavior variations.

**Figure 6:**
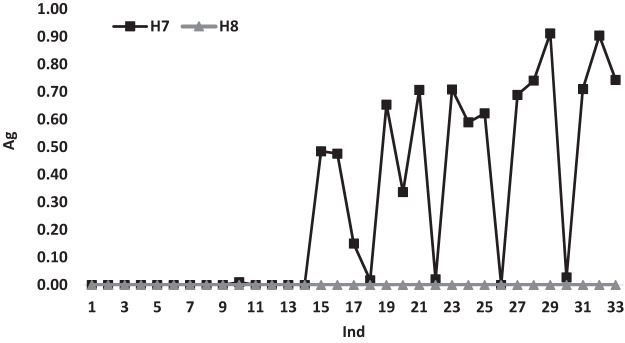
The average gap for *H*_7_ and *H*_8_ according to *Ind*.

Figure 6 illustrates the variations of the average gap for heuristics *H*_7_ and *H*_8_ according to *Ind*. Both heuristics show a similar variation behavior with a gap value of 0.00 till *Ind* = 14m after this point the variations changed for *H*_7_ and has different gap values reaches to 0.90 at some points, while the gap variations of *H*_8_ continues to have a 0.00 gap values for all *Ind*, which shows the dominance performance of *H*_8_ over all the used heuristics.

The results presented above, indicate that the developed heuristics were capable to derive an accepted approximate solution with a good execution time, but none of the given heuristics were capable to produce exact or optimal solution, which requires more work and research to enable these heuristics to produce optimal solutions for the given problem. Besides, the algorithms presented by Taha, Ould-Slimane & Talhi (2020) can inspire these heuristics to develop new algorithms for the studied problem.

Moreover, the developed heuristics can be used as an initial solution for other meta heuristics, such as, genetic algorithm, particle swarm optimization algorithms to enhance the given solution.

The performance of the developed heuristics was compared across each other, since to the best of our knowledge this problem was not studied previously in any of the literature that was within our reach.

Other type of instances regarding the big scale of number of reports. Indeed, for this type of instances the number of reports *n*_*r*_ will be in {50, 100, 200, 500} and the number of doctors *n*_*d*_ will be in {3, 5, 10, 15}. Therefore, we generate 800 instances of this type. The overview of the proposed heuristics according to *Perc*, *Ag* and *Time*, is shown in Table 8.

**Table 8:** Overview of heuristics according to *Perc*, *Ag* and *Time* for the big scale instances.

	*H*_1_	*H*_2_	*H*_3_	*H*_4_	*H*_5_	*H*_6_	*H*_7_	*H*_8_
*Perc*	44.4%	0.0%	6.0%	10.9%	28.6%	28.1%	28.3%	98.0%
*Ag*	0.41	0.90	0.88	0.82	0.63	0.64	0.63	0.02
*Time*	0.000	0.000	0.036	0.049	0.209	0.302	0.291	0.068

Table 8 shows that the heuristics *H*_1_, *H*_2_ and *H*_8_ keep the same range of percentages comparing with the small instances illustrated in Table 4. However, for the other heuristics the percentages range are remarkably increased. Indeed, for *H*_3_ the percentage for the small instances is 48.1% (see Table 4), while for the big scale *Perc* = 6%. The *H*_8_ heuristic is always the best, with 98%.

## Conclusion

This work presented the solution to the problem of paper workload distribution between doctors in healthcare organizations to ensure fair distribution of the overall workload based on the total number of pages of patients’ reports assigned to each doctor. This problem was solved by developing eight heuristics based on dispatching rules with several variants, randomization approach, clustering methods, probabilistic method and iterative methods approach. The performance of the developed heuristics was measured experimentally based on different classes with 2,450 instances. The obtained results showed a clear variation in the performance measurements of the developed heuristics based on the elapsed time and the gap value calculations. The given results showed that heuristic *H*_8_ was dominant among all the developed heuristics with remarkable performance and 0.00 gap values for all the used instances. For future work, the developed heuristics can be adopted in other domains like education, finance, industry, aviation, and many others. Also, it can be used in branch and bound for exact methods.

## Supplemental Information

10.7717/peerj-cs.819/supp-1 Supplemental Information 1 Raw data. The generated classes are as follows: Class A: *n_p_*j ∈ U(10, 20). Class B: *np_j_* ∈ U (20, 30). Class C: *np_j_* ∈ U (10,30). Class D: *np_j_* ∈ N(20, 5). Class E: *np_j_* ∈ N(20, 10).

10.7717/peerj-cs.819/supp-2 Supplemental Information 2 Code in C++ for LPT.

## Additional Information and Declarations

### Data Availability

The following information was supplied regarding data availability: Raw data and code are available in the Supplemental Files.
